# Perinatal Gram-Positive Bacteria Exposure Elicits Distinct Cytokine Responses In Vitro

**DOI:** 10.3390/ijms22010332

**Published:** 2020-12-30

**Authors:** Edith Reuschel, Martina Toelge, Sebastian Haeusler, Ludwig Deml, Birgit Seelbach-Goebel, Maria Emilia Solano

**Affiliations:** 1 University Department of Obstetrics and Gynecology At The Hospital St. Hedwig of The Order of St. John, University of Regensburg, 93049 Regensburg, Germany; Sebastian.Haeusler@barmherzige-regensburg.de (S.H.); Seelbach.B@web.de (B.S.-G.); 2 Institute of Clinical Microbiology and Hygiene, University Hospital Regensburg, 93053 Regensburg, Germany; Martina.Toelge@klinik.uni-regensburg.de (M.T.); Ludwig.Deml@lophius.com (L.D.); 3 Department of Obstetrics and Feto-Maternal Medicine, University Medical Center Hamburg-Eppendorf, 20246 Hamburg, Germany

**Keywords:** preterm birth, gram-positive bacterial infection, neonatal immunity, sex-specific immunity

## Abstract

During pregnancy, infections caused by the gram-positive bacteria *Enterococcus faecalis* (*E. faecalis*), *Streptococcus agalacticae* (*S. agalacticae*), and *Staphylococcus aureus* (*S. aureus*) are major reasons for preterm labor, neonatal prematurity, meningitis, or sepsis. Here, we propose cytokine responses to bacterial infections by the immature perinatal immune system as central players in the pathogenesis of preterm birth and neonatal sepsis. We aimed to close the gap in knowledge about such cytokine responses by stimulating freshly isolated umbilical blood mononuclear cells (UBMC) with lysates of *E. faecalis*, *S. agalacticae*, and *S. aureus* collected from pregnant women in preterm labor. Bacterial lysates and, principally, *S. aureus* and *S. agalacticae* distinctly triggered most of the eleven inflammatory, anti-inflammatory, TH_1_/TH_2_ cytokines, and chemokines quantified in UBMC culture media. Chemokines depicted the most robust induction. Among them, MIP-1β was further enhanced in UBMC from female compered to male newborn infants. Due to its stability and high levels, we investigated the diagnostic value of IL-8. IL-8 was critically upregulated in cord blood of preterm neonates suffering from infections compared to gestational age-matched controls. Our results provide novel clues about perinatal immunity, underscoring a potential value of IL-8 for the timely detection of infections and suggesting that MIP-1β constitutes an early determinant of sex-specific immunity, which may contribute, e.g., to male’s vulnerability to preterm birth.

## 1. Introduction

Prematurity due to preterm birth is a main cause of short-term (e.g., respiratory distress and intraventricular hemorrhage) or long-term neonatal morbidities [1] and even death. Worldwide, 15 million babies per year are born due to preterm labor [2] arising from uterine contractility prior to 37 weeks of gestation in human [3]. This early onset of labor has been associated to multiple factors, among which intrauterine inflammation and ascending genital tract infections play a leading role [4]. It is typically considered that intrauterine infections induce the release of potent inflammatory mediators, such as cytokines and chemokines, which promote leukocyte recruitment and inflammation and ultimately result in premature uterine contractions and birth [5]. Intriguingly, these inflammatory reactions are mediated not only by maternal uterine leukocytes but also by fetal immune cells that migrate to the uterus and are highly reactive towards environmental signals [6,7].

High frequencies of pathogenic gram-negative and -positive bacteria were identified in vaginal and cervical swabs of women with impeding preterm labor [8]. The most ubiquitous species was the gram-negative *Escherichia coli*, which was present in 90% of the women investigated. Gram negative bacteria, often of nosocomial origin and known to cause dangerous infections, have shown concerning increases in antimicrobial resistance over the last decades [9]. Remarkably, the gram-positive *Enterococcus faecalis* (*E. faecalis*), Group B Streptococcus (GBS), and *Staphylococcus aureus* (*S. agalactiae*) were also highly prevalent, being respectively detected in 64%, 24%, and 10% of women with impeding preterm birth [8]. Although these three gram-positive bacteria species are often present in the vaginal microbiome [10], they can also mediate opportunistic perinatal infections, chorioamnionitis, and/or neonatal sepsis, causing a substantial disease burden to Intensive Care Units [11].

In fact, an increase in the incidence of enterococcal bacteremia frequently of nosocomial origin has been reported, with *E. faecalis* being the most prominent strain [12,13,14]. *E. faecalis* does not account for a large number of neonatal sepsis cases, but it can be responsible for infections of the urogenital tract and endocarditis both in the mother and the neonate [15,16]. In turn, the gram-positive GBS *Streptococcus agalactiae* (*S. agalacticae*), harmless in pregnant women, can also cause severe infections in neonates. Despite intrapartum antibiotic prophylaxis, *S. agalactiae* is still the pathogen that most frequently causes early-onset sepsis within the first week of life and invasive GBS infection continues to be the leading cause of pneumonia and meningitis in neonates [15,16]. Finally, *S. aureus* is an emerging cause of chorioamnionitis and preterm rupture of membranes. Infants born from mothers positive for these bacteria present increased odds of *S*. *aureus* colonization [17], which is a risk factor for subsequent *S. aureus* infection. Such infections are among the most common causes of late-onset sepsis, for example, in very-low birth weight neonates [18,19].

Taken together, these vaginal pathogenic bacteria not only pose a serious threat for the baby to be born too early but also may lead to congenital infections in the uterus, at birth, or after a latency period [20,21]. This may occur due to, e.g., aspiration or ingestion of bacteria in the amniotic fluid or during passage through the birth canal [22]. Due to immaturity of the neonatal immune system, such an infection can lead to sepsis [23]. Neonatal sepsis is a systemic condition that elicits hemodynamic changes, tissue damage, and distinct clinical features. As some of these symptoms can be provoked by potent inflammatory cytokines, neonatal sepsis has also been referred to as systemic inflammatory response syndrome [22]. Despite progress in perinatal care, the incidence of prenatal and neonatal sepsis remains approximately two per 1000 live births, constituting a significant hardship at these early life stages. Further improvements are still required for its timely diagnosis, as studies have shown that 60 percent of lethal neonatal sepsis failed to be identified prior to death [23,24]. In this context, forthcoming challenges appear to surface in obstetric and neonatal care with regards to perinatal infections. Indeed, the continuous increase in antimicrobial resistance requires not only early detection of infections but also availability of antimicrobials with higher efficacy, i.e., by using superior host materials, to control them [25].

Based on the evidence reported, it is tempting to hypothesize that the cytokine responses by perinatal leukocytes to bacterial infections are a fundamental element in the pathogenesis of preterm birth and neonatal sepsis. Here, we postulate that bacteria present in the vagina of women undergoing preterm labor induce distinct inflammatory responses. Given that perinatal *E. coli* infection has already been the subject of thorough investigations [26], the aim of our present study was to characterize in depth the cytokine responses specific to the three clinically relevant vaginally occurring gram-positive bacteria frequently found in pregnant women with preterm labor, namely *S. agalacticae*, *E. faecalis*, and *S. aureus.* We hypothesize that these bacteria evoke distinct cytokine and chemokine responses that not only pose critical clinical challenges but also may hold diagnostic value.

To test this hypothesis, we first established an in vitro system of freshly prepared umbilical cord blood mononuclear cells (UBMC) from uncomplicated term deliveries, which we stimulated with the lysates of the mentioned gram-positive vaginal bacteria. The release of relevant preselected cytokines and chemokines upon bacterial stimulation was determined using the Luminex technology. The existence of sex-specific patterns in cytokine secretion was assessed. Based on these findings, IL-8 was proposed as a stable and reliable diagnostic marker for perinatal infections and was tested in cord blood of preterm neonates. 

## 2. Results

### 2.1. Bacterial Lysates Trigger the Secretion of Cytokines and Chemokines by Umbilical Cord Blood Mononuclear Cells

**Figure 1:**
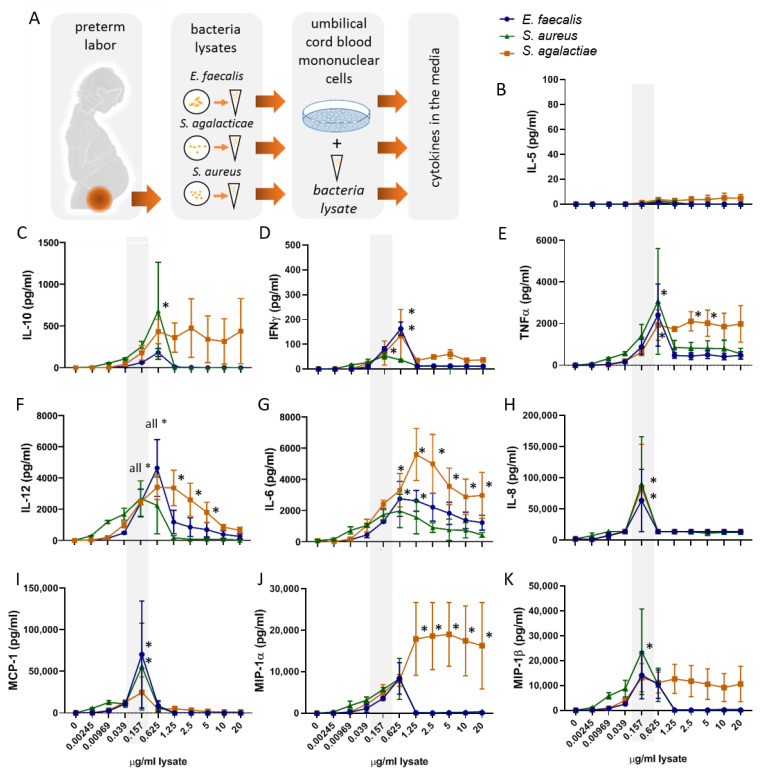
Experimental setup and optimization of umbilical blood mononuclear cells and bacteria lysate stimulation conditions: in our experimental design (**A**) vaginal and cervical bacteria samples from women with impeding preterm labor were collected. *E. faecalis*, *S. agalacticae*, and *S. aureus* were isolated and cultured to produce bacteria lysates. Umbilical blood mononuclear cells (UBMC) from ten term-born infants were stimulated in triplicates for 36 h with or without the respective bacterial lysates. The levels of preselected cytokines and chemokines were determined in the supernatants. (**B**–**K**) Increasing concentrations of bacteria extract (0.00245–20 µg/mL lysate) were tested for the induction of ten cytokines (IL-5, IL-10, IFNγ, TNFα, IL-12, IL-6, IL-8, MCP-1, MIP-1α, und MIP-1β), detected using luminex technology. With exception of IL-5 (**B**), the bacteria lysates significantly induced cytokine secretion (**C**–**K**) (two-way ANOVA *p* < 0.05). * *p* < 0.05 in Dunnett’s multiple comparisons test. Concentrations of 0.157 µg lysate/mL (grey shadow) were selected for further experiments. IL-12 refers to IL12p40/70. The three bacteria are depicted as follows: *E. faecalis* (blue circles), *S. agalacticae* (orange squares), and *S. aureus* (green triangles).

Here, we established an in vitro setting in which UBMC freshly isolated from healthy neonates born at term were incubated for 36 h with the lysates from the selected gram-positive vaginally occurring bacteria found in vaginal swabs of pregnant women with impending preterm birth: *S. agalacticae*, *E. faecalis*, and *S. aureus* (Figure 1A).

We first aimed to identify the optimal bacterial lysate concentrations among the ten dilutions tested as well as to select the cytokines of relevance in the context of the studied bacteria (Figure 1). Here, the cumulative release of cytokines upon 36 h of bacterial lysates stimulation was analyzed by Luminex in the conditioned culture media of UBMC. As shown in Figure 1C–K, a bimodal dose-dependent induction of most of the pro-and anti-inflammatory, and TH_1_-type cytokines and chemokines investigated was observed. Specifically, all cytokines rose between 0.0039 and 0.625 µg/mL of the bacterial lysate, whilst higher concentrations resulted in either stabilization or reduction of cytokine values. We selected the concentration of 0.157 µg/mL of the gram-positive lysate as optimal to detect changes in the cytokines and chemokines of interest. By selecting this concentration, we ensured that the immune response remained operational and below toxicity or saturation levels, which may otherwise prevent the assessment of differences between the effects of the three bacteria lysates. In this regard, the release of TNFα and IL-12 in the culture media was also compared between samples stimulated for 4 or 36 h. As expected, at 4 h, the secretion of the pro-inflammatory cytokine TNFα, associated to initiation of the immune response, was already comparatively high, whereas the levels of the cytokine IL-12, involved in promoting a TH_1_-type response, remained close to detection levels, mirroring the expected delay of adaptive immune responses (Figure S1A,B). After 36 h of bacteria stimulation, the cytokine levels were detectable and still increased, indicating that this is an adequate time to evaluate both innate and adaptive cytokine responses in our in vitro system.

Of note, bacterial lysates did not have a significant effect on the levels of IL-5 (Figure 1H) and TGFβ (Figure S2A) (two-way ANOVA analysis, factor: lysate concentration *p* > 0.1). Additional determinations confirmed that these culture conditions do not result in significant IL-5 secretion (Figure S2B). For these reasons, IL-5 and TGF-ß were not included in further analyses.

### 2.2. The Bacterial Lysates Elicit Distinct Patterns of Cytokines Responses

In a next step, ten UBMC samples were stimulated with 0.157 µg/mL of bacterial lysate or with culture media (negative control) for 36 h. Relevant information about the UBMC donors is summarized in Table 1. Levels of secreted anti-inflammatory (IL-10), inflammatory or TH_1_-cytokines (IL-6, TNFα, IL-12, and IFNγ), as well as inflammatory chemokines (IL-8, MIP-1α, MCP-1, and MIP-1β) were measured by Luminex.

**Figure 2:**
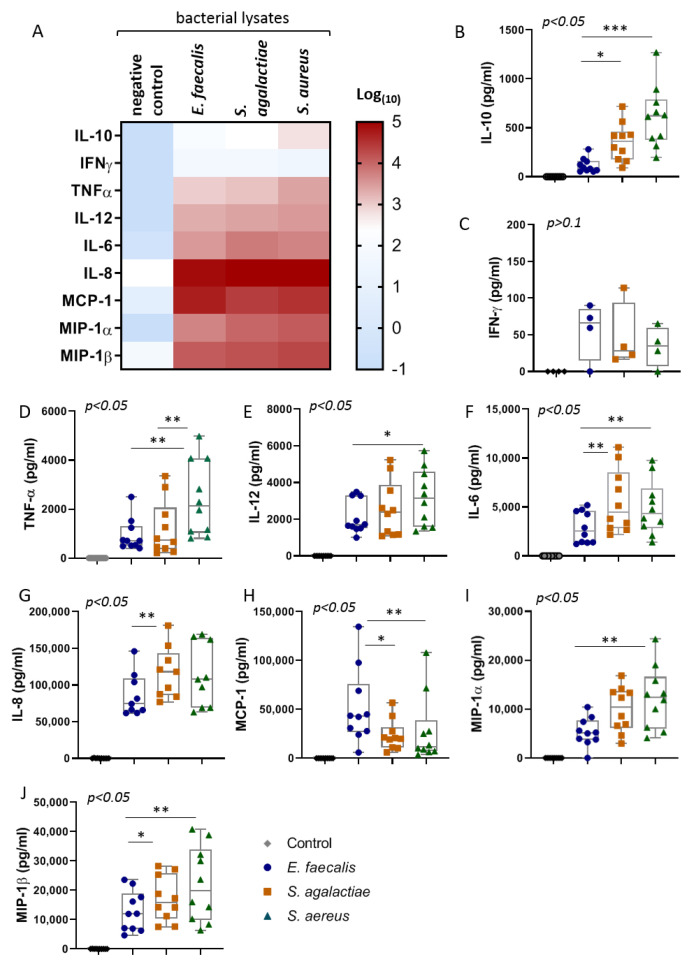
The bacterial lysates elicit distinct patterns of cytokines responses. (**A**) The heatmap depicts the median value for the cytokines and chemokines indicated in the left (rows), according to the bacterial stimuli (columns). The color scale is defined on the right. As the median values are reported in logarithmic (10) scale, values of 0 pg/mL were replaced by 0.1 pg/mL. (**B**–**J**) Boxplots for each one of the cytokines and chemokines analyzed upon stimulation with or without lysates from *E. faecalis* (circles), *S. agalacticae* (squares), *S. aureus* (triangles), and controls (diamonds). Overall statistical differences in the cytokine levels between the three bacteria lysate stimulations were compared using the nonparametric Friedman Test (*p* values are presented in the top left of B–J). *p* values for the posterior multiple comparisons are depicted as * *p* < 0.05; ** *p* < 0.001; *** *p* < 0.0001 (Dunn’s Test correction). *n* = 10. IL-12 refers to IL12p40/70. Boxplots represent the median, minimum, and maximal values, whilst the dots depict the individual values.

As expected, compared to the negative controls, the three prepared bacterial lysates (*S. agalacticae*, *E. faecalis*, and *S. aureus*) potently induced the secretion of all nine cytokines and chemokines included in this analysis (Figure 2A). The qualitative evaluation of the heatmap for all cytokines according to the bacterial lysate employed suggested a generally stronger cytokine response in the case of *S. aureus* than the other bacteria. We confirmed this differential response of the UBMC to the bacterial lysates by comparing the levels of each cytokine or chemokine using Friedman test for paired samples. In particular, significantly higher levels of IL-10, TNFα, IL-12, IL-6, MIP-1β, and MIP-1α were detected in cultures stimulated with *S. aureus* than in those with *E. faecalis* lysates (Figure 2B–J). In turn, *S. agalactiae* evoked intermediate responses, with higher levels of IL-10, IL-6, IL-8, and MIP-1β than *E. faecalis* and lower levels of TNFα than *S. aureus.* In contrast, an opposite trend was observed in the case of MCP-1, which presented higher levels in *E. faecalis* than in the other bacteria lysate stimulation (Figure 2H). Finally, no significant differences were found among the minor secretion of IFNγ triggered by the three bacterial lysates (Figure 2C).

Noteworthy, in all stimulation conditions, IL-8 was the cytokine that reached the highest concentrations in the culture media. These findings underscore that, among the detected cytokines, IL-8 may represent the most sensitive biomarker for cord blood diagnosis of perinatal infections with these three bacterial species.

### 2.3. Umbilical Blood Mononuclear Cells from Female Infants Respond More Pronouncedly to Bacterial Lysates than Those from Male Infants

**Figure 3:**
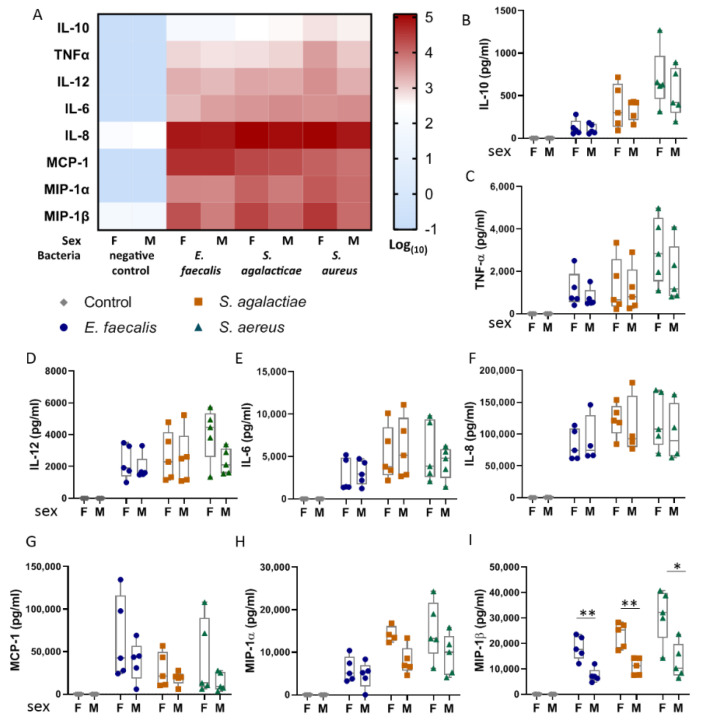
The sex of the infant determines the intensity of MIP-1β responses to bacterial lysates. (**A**) The heatmap presents in each row the median value for the cytokine and chemokines depicted in the left according to the treatment and the sex of the infants from whom the samples were collected (columns). The values are presented in logarithmic (10) scale as shown in the right. (**B**–**I**) The individual cytokines and chemokines detected upon stimulation with or without lysates from *E. faecalis* (circles), *S. agalacticae* (squares), *S. aureus* (triangles), and controls (diamonds) are presented in boxplots. For each bacteria lysate, the levels of the respective cytokines were compared between cultures of UBMC from female (**F**) and male (**M**) donors: * *p* < 0.05; ** *p* < 0.001. MannWhitney U Test. Boxplots represent the median, minimum, and maximal, whilst the dots depict the individual values.

In order to dissect possible factors that influence the immune response to the respective bacteria, we next considered the cytokine response to the bacterial lysates according to the sex of the infants who donated the UBMC samples. In the cultures used as negative controls, no differences in the basal cytokine levels were observed between females and males (Figure 3A). The qualitative assessment of the heatmap patterns (Figure 3A) indicated that samples derived from female infants presented a widespread more robust response to bacteria lysates than UBMC derived from male infants. In particular, the strongest sex dimorphism was observed in the levels of the chemokine MIP-1β in response to the three bacteria lysates (Figure 3I), whereas a similar but nonsignificant trend was observed in the cases of MCP-1 and MIP-1α (Figure 3G–H).

Of note, the sex of the sample donors had no influence on the levels of IL-8, suggesting that this chemokine constitutes a stable marker to screen for neonates suffering from infections at birth, independent not only of the infecting bacteria species but also of the sex of the neonate.

### 2.4. IL-8 Levels in Cord Blood of Preterm Newborn Infants is a Stable Indicator of Perinatal Infections

**Figure 4:**
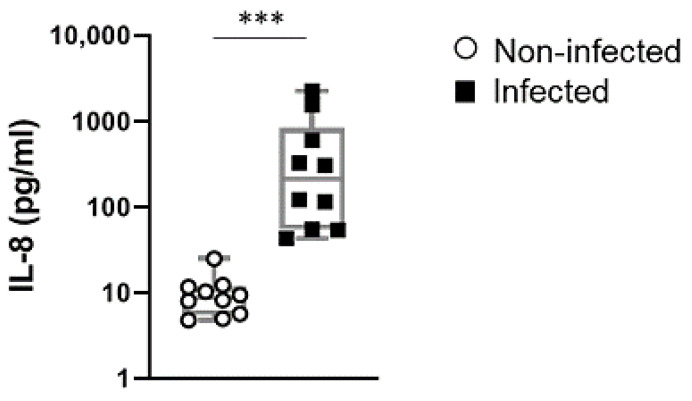
IL-8 was significantly increased in preterm newborns that suffered from infections. The levels of IL-8 were determined in cord blood of preterm born infants that suffered (back squares) or not (white circles) from infections. The results are presented in boxplots indicating the median, percentiles, and maximum and minimum values, whilst the dots depict the individual values. *** *p* < 0.0001, Mann–Whitney U Test. *n* = 10.

In a next step, we investigated whether IL-8 was also detectable in infants suffering from infections in vivo. In this analysis, we collected cord-blood samples from newborn infants that were born preterm suspected to be due to infections. Cord blood collected from gestational age-matched infants born due to noninfectious pregnancy pathologies served as control (Table S2). As depicted in Figure 4, we observed that the levels of IL-8 dramatically increased in the cord blood of neonates that were confirmed to suffer from bacterial infections, compared to those not affected by infections. These results suggest that IL-8 may constitute a useful marker to rapidly detect neonates affected by infections at birth.

## 3. Discussion

In the presence of perinatal infections, the triggered cytokine milieu is critically involved in the progress and outcome of the immune response. We could observe here that freshly isolated UBMC responded specifically to in vitro stimulation by lysates of three common vaginal gram-positive bacteria species. This involved a substantial upregulation of the chemokines IL-8 and MCP-1, as well as MIP-1α and MIP-1β by the bacterial lysates. In the context of intraamniotic infection, these chemokines establish a gradient that favors the migration of neutrophils and other effector leukocytes from the maternal or fetal circulation into the chorioamniotic membranes or umbilical cord, respectively [27].

Strikingly, MIP-1β secretion presented a marked sex-specific pattern, characterized by reduced levels in the case of male UBMC donors. This appeared as a solid phenomena, which manifested with the three bacteria studied. In line with our observations, the male sex of the offspring is a well-known risk factor for preterm birth as well as for neonatal complications [28]. This includes the higher vulnerability of males to severe neonatal infections and sepsis [29] in contrast to a sex bias towards more efficient immune responses in females, that endures throughout life [30]. Clearly, it would be interesting to learn if this sexual dimorphism is swiftly detectable in vivo. However, as shown by our data and others, the diverse bacteria and times postinfection [31] are factors that cause significant variability in MIP-1β and that cannot readily be controlled for in human infections, likely hindering the recognition of sex differences in MIP-1β secretion in the context of perinatal infections. Hence, besides the current ex vivo experiments, further clues about the importance of MIP-1β in sex-specific responses may be gained using mouse models of perinatal infection, e.g., with *S. aureus*. Indeed, contrasting reports appear in the literature comparing sex-specific expression of chemokines in the context of host response or inflammatory tissue damage [30,32,33], which could be explained by the disparity in the progress of the immune responses analyzed. For example, MIP-1β was increased by LPS in the blood [34] and brain [35] of female but not of male mice. However, the characterization of sex-specific responses to gram-positive bacteria remains to date elusive and merits further investigations. Intriguingly, due to its potent chemoattractant capacity on Treg cells [36], MIP-1β-increased secretion by cells from females might be involved in a more efficient resolution of the inflammatory response and the prevention of neonatal sepsis compared to male neonates. Hence, we propose that the reported robust upregulation of the chemokines MIP-1β at early stages of the inflammatory response in females may enhance a more rapid immune response than in males and may provide efficient host defense, consequently restraining propagation of the infection to reach a posterior sepsis state. In this manner, the studied chemotactic signals may constitute key early events driving sex specificity in the pathogenesis of infection-associated preterm labor and perinatal infections.

Although it is generally considered that sexual dimorphism in immunity responds chiefly to female or male sex hormones, it may also be influenced by genetic or immune traits inherent to each sex [33,37]. Our reported sex-specific differences in MIP-1β secretion in the minimalist design of our in vitro system can only stem from intrinsic differences between the UBMC responses in females and males. For example, leukocytes from females have been reported to express greater density of toll-like receptors, critically involved in pathogen recognition [30], which could be associated to the induction of stronger responses. Although critical mechanisms underlying sex differences in acute inflammation remain unknown, our observations could be also related to subtle changes in the immune function of the UBMC, which is the subject of our current studies.

In comparison to the other investigated chemokines, IL-8 presented no sex-dimorphism and exhibited the most robust release throughout our experiments. The low variability and high levels reached by IL-8 suggested that it could be readily detected in cord blood from infants suffering from perinatal infections. Previously, neonatal serum IL-8 in combination with clinical anamnesis has been proposed as a helpful biomarker for early diagnosis of neonatal sepsis [38]. Indeed, we could confirm exacerbated IL-8 levels already in cord blood of preterm neonates suffering from intrauterine infections, which was unambiguously increased compared to non-infected neonates. The high levels reached by IL-8 could be an advantage to establish clinically applicable threshold values, indicating that IL-8 could hold high relevance as a diagnostic biomarker for early identification of infections in the neonate. Noteworthy, IL-6 and IL-10 have also been recommended as indicators of neonatal sepsis [39] and increased levels of IL-6 and TNFα in vaginal secretions of women with preterm premature rupture of membranes were good predictors of fetal inflammatory response syndrome [40]. Hence, further studies are required to unequivocally compare the relevance of these cytokines in diverse clinical settings within the perinatal period. Moreover, advances in clinical methods for bacteria detection, for example, the optimization of Bioprinted Microarray Based Interferometric Point-of-Care Device [24], may provide in the near future helpful tools to rapidly identify infections in the perinatal clinical diagnosis setting.

IL-8, also called CXCL8, is a pro-inflammatory chemokine classically secreted by monocytes, macrophages, and endothelial cells in adulthood to recruit and activate neutrophils as well as γδ T cells [34]. During pregnancy, intrauterine IL-8 secretion in response to toll-like receptor stimulation is considered an upstream event eliciting preterm birth [5]. Dudley et al. [41] showed that decidual cells produced significant amounts of IL-8 among other chemokines in response to intact GBS in a strain-specific fashion. In our work, the enhanced response in preterm neonates suffering from infections underscores a role for IL-8 as a fetal signal to trigger labor in the presence of intrauterine infection. Intriguingly, in term and preterm human newborns, IL-8 is released not only by innate cells but also profusely by cord blood T cells [42]. Such exacerbated IL-8 responses possibly compensate for the incapacity of the neonatal immune system to mount proper TH_1_ responses due to deficient IL-12 production by early life dendritic cells [42]. IL-12 deficiency is casually involved in perinatal vulnerability to infections for example by GBS [43], as administration of IL-12 to neonatal mice infected with GBS improves bacterial clearance and survival, highlighting the importance of IL-12 in resolving neonatal gram-positive sepsis [44]. From our present data, it is not possible to assert whether IL-12 release was deficiently induced in cord blood cells, and we observed comparable IL-12 levels among the three bacteria lysates studied. IL-12 can stimulate naïve T-cells to differentiate into TH_1_ cells, hereby enhancing the production of IFNγ and cell-mediated immunity [40]. In our in vitro system, IFNγ was only marginally induced in all cultures, reinforcing a limited ability of UBMC to mount TH_1_ responses also upon stimulation with *E. faecalis*, *S. agalactiae*, and *S. aureus* components.

Moreover, we could confirm our hypothesis that lysates of different gram-positive bacterial species induce distinct inflammatory responses in terms of the soluble markers investigated. These bacteria contain individual, strain-specific compositions of pathogen-associated molecular patterns (PAMPs) such as peptidoglycan monomers, lipoteichoic acids, mannose-rich glycans, lipoproteins, flagellin, and CpG motif containing DNA among others. Binding of these components to pattern recognition receptors (PRR) triggers in a co-ordinate activation of different effector cells of the innate immune system, resulting in the production of various cytokines and chemokines [45,46]. Herein, unique compositions of immune-modulatory components within the lysates of tested bacteria may trigger the release of the observed strain-specific profiles of cyto-/chemokines by UBMC. In our in vitro setting, we could observe that *E. faecalis* lysates generally elicited more moderate response than *S. agalactiae* and *S. aureus*. In fact, despite its pathogenicity, *E. faecalis* has been postulated as a potential probiotic [47]. The maternal *E. faecalis* colonization of the gastrointestinal tract in healthy newborns has been associated to protection from gut infections in the later life of the infant [48]. It has been reported that *E. faecalis* produces antibacterial compounds and attenuates inflammatory responses to other pathogens in vitro and in vivo, e.g., by suppressing IL-8 or by promoting IL-10 secretion [47], which together may account for the relatively moderate cytokine stimulation observed here in response to *E. faecalis.* An exception to this is MCP-1, which was prominently induced by *E. faecalis* lysates compared to lysates form the other bacteria. MCP-1, also called CCL2, mediates the recruitment of monocytes, activated T-cells, basophils, NK-cells, and immature dendritic cells. MCP-1 secretion by neutrophils can prevent severe sepsis stemming from oral *E. faecalis* infection [49] by modulating macrophage differentiation. Importantly, a potent MCP-1 response appears as a salient trait in response to *E. faecalis* infection, which has been referred to by in vitro and in vivo studies [50] and may potentially serve to differentiate *E. faecalis* from other neonatal infections.

Although *S. agalactiae* is often part of the gastrointestinal and genitourinary microbiota in immunocompetent individuals, it is also a leading cause of maternal chorioamnionitis, neonatal meningitis, and sepsis worldwide. Our results show that *S. agalactiae* induced prominent inflammatory responses in UBMC, indicating that the neonatal immunity is readily responsive to these bacteria components. Still more potent was the response of UBMC to *S. aureus* lysates, a commensal bacterium that is among the most common causes of chorioamnionitis, preterm birth, and late-onset sepsis in very-low birth weight infants [19]. Rather than stimulating MCP-1, *S. agalactiae* and *S. aureus* elicited higher MIP-1α release than *E. faecalis*. MIP-1α participates in the host response, e.g., to invading bacteria by regulating the trafficking and activation state of leukocytes such as macrophages, lymphocytes, and NK cells [51,52]. The stepwise upregulation of MIP-1α first by *S. agalactiae* and then by *S. aureus* respectively may relate to an enhanced inflammatory profile, also mirrored in a corresponding stepwise increase of TNFα and the anti-inflammatory cytokine IL-10 after incubations with *S. aureus* lysate. IL-10 is an important regulator and suppressor of the pro-inflammatory cascade [53]. It is produced at the maternal–fetal interface to promote immune tolerance of the fetal allograft [53]. Also, administration of IL-10 increases the survival of neonatal mice suffering from GBS sepsis [54] due to its potential to restrict inflammation and to reduce tissue damage in the host defense [55]. Moreover, *S. aureus* lysates elicited the highest secretion of TNFα, indicating robust activation of the innate immunity in these cultures [56,57]. It remains however unclear why lysates of *S. aureus* show a stronger capacity than the lysates of the other bacteria to trigger the release of most cytokines by UBMCs. Also in the case of *S. aureus*, recent research has shown that bacterial wall compounds including lipoproteins [58] but not peptidoglycans [59] are centrally required for immune responses elicited in the context of arthritis. Indeed, *S. aureus* lipoproteins potently target macrophage TLR2 to induce chemokines secretion and to either trigger septic arthritis (if lipoproteins were administered alone) or to strengthen the clearance of bacteria in the case of infection [58]. In the case of *S. agalactiae* among the many components proposed to play a role [60], lipoproteins are important mediators of its virulence [61]. Hence, it remains to be investigated whether the observed exacerbated effects of the *S. aureus* over the other bacteria are mediated by distinct properties of each bacteria lipoproteins and whether, in the context of perinatal infections, lipoproteins also may mediate a comparable effect to arthritis, e.g., by potentiating chemokine secretion and the resolution of infection.

Finally, with the present data, we have advanced our previous work [26,62] by the laborious optimization of our in vitro model by testing multiple dosages of lysates of clinically relevant gram-positive bacteria collected in our clinic and by analyzing comparatively the evoked responses. Indeed, UBMC stimulations proved to be an efficient noninvasive method to assess neonatal immunity to unveil distinct profiles of cytokine responses in neonates. Importantly, in our settings, cytokine secretion evoked by increasing levels of bacteria lysates presented a bimodal response, with stabilization or reduction of cytokine levels in stimulations with higher concentrations of bacteria lysates. Such a bimodal response has been already reported in multiple settings [63,64,65,66,67]. Suppression of cytokine production in high stimulatory conditions may rely on auto/paracrine and intracellular negative feedback pathways [63,68]. Alternatively, high concentrations of bacterial products, such as LPS, can trigger excessive oxidative mediators with toxic effects and can reduce cell viability [65,66]. For these reasons, in the optimization, we decided to favor an intermediate bacterial lysate concentration, which evoked significant cytokine responses that remained below toxicity or saturation levels.

We consider that the present observations provide valuable insights in the capacity of the studied bacteria to activate and modulate the perinatal immune system and thus to contribute to a rational estimation of the importance of these pathogens in pregnancy and early life. In the present study, we prioritized screening for the cumulative secretion throughout 36 h of stimulation, as an indicator of the type and potency of the overall inflammatory responses evoked by the three bacteria. Within this time frame, a substantial immune activation is expected to take place, as evidenced by the rising levels of TNFα and IL-12. This approach is possible given that our [69,70] and other [71] previous work showed that the detected cytokines present sufficient stability for at least 36 h in the present culture conditions at 37 °C. The only exception to this prolonged stability may reside in TNFα levels [70], for which, although the presented comparisons remain valid, they may not represent the full cumulative TNFα induction throughout the complete stimulation period. Noticeably, our study triggers interesting questions about the dynamics of the inflammatory response to different bacteria, which due to its high relevance in the context of perinatal infections and neonatal sepsis shall be investigated in upcoming studies.

Importantly, in the present work, we could confirm our in vitro findings also in the perinatal responses in vivo by reporting enhanced IL-8 secretion in preterm born infants suffering from infections, which underscores the clinical application of our observations. Our data of sex-specific differences in the secretion of chemokines, particularly of MIP-1β at early stages of the immune response, may contribute to shedding light on as yet undiscovered mechanisms underlying male vulnerability to perinatal complications [37], which merits further examination. We consider that our experimental method opens new opportunities for investigations, for example, of the responses of UBMC from preterm vs. term born neonates. It also gives new possibilities for translational research, for example, by contrasting our findings with the levels of cytokines in women at risk from preterm birth and by evaluating their risk of infection-related complications after birth.

Taken together, we envision that the present research advances towards the ultimate goal of developing strategies to prevent, identify, and treat infection-triggered preterm birth and the associated complications of mothers and babies.

## 4. Materials and Methods

### 4.1. Umbilical Cord Blood Source

Informed consent was voluntary obtained either from twelve healthy mothers or from women undergoing preterm birth. Healthy participants that delivered vaginally at term were nonsmokers, were not under medical treatment, and showed no signs of infection during pregnancy. In the case of cord blood samples collected from preterm-born infants, control samples were collected from participants undergoing preterm delivery due to noninfectious conditions, such as preeclampsia including severe hypertension in pregnancy, and placenta insufficiency followed by fetal growth retardation. Additional samples collected from neonates born preterm, suspected to be due to infections, were included in the study after prospective confirmation of the presence of bacterial infections.

Following ligation of the umbilical cord, cord blood was collected immediately at birth in citrate bags, stored at 4 °C in the fridge, and processed within 12 h for in vitro *studies* (Table S1). For detection of IL-8, plasma from preterm born infants was isolated, transferred to the lab and measured using a commercial Elisa. The protocol of the study was conducted in accordance with the World Medical Association Declaration of Helsinki and approved by the Ethical Commission of the University of Regensburg, Center for Clinical Studies (Approval number 06/098, date of approval 9 August 2006, last amendment 23 January 2015).

### 4.2. Isolation of Umbilical Cord Blood Mononuclear Cells (UBMC)

The freshly citrated umbilical cord blood was diluted 1:3 with PBS. Subsequently, 25 to 30 mL of the diluted blood were carefully overlaid by 15 mL Pancoll (Lymphocyte sep. medium/Pancoll human, density 1.077 g/mL, PAN, Biotech, Aidenbach, Germany) in a 50-mL tube. UBMC were separated by density gradient centrifugation (800× *g*, 30 min) at room temperature (RT), harvested as a single interface layer between the Pancoll cusion and the blood plasm, and washed three times with 45 mL PBS. Contaminating erythroblasts were eliminated by lysis in 1-mL erythrocyte lysis buffer (BD Pharm Lyse^TM^ Lysing Buffer, Cat. No. 555899, Becton Dickinson Biosciences Pharmingen, San José, CA, USA) for 10 min at RT, followed by two additional washes in 45 mL PBS. Cell pellets of each donor were resuspended in T-cell medium (RPMI 1640 medium, Gibco, Paisley, Scotland), supplemented with 10% heat-inactivated (1 h, 56 °C) human AB serum, and enumerated using a Neubauer counting chamber to determine the total number of cells. Total cell number was adjusted to 2 × 10^5^ cells per 90 µL in T-cell medium.

### 4.3. Preparation of Bacterial Lysates

Each clone of the Streptococcus, Enterococcus, and Staphylococcus strains was isolated from a vaginal swap of a 29-year-old patient suffering from PTL at the Department of Bacteriology, Institute of Clinical Microbiology and Hygiene, University Hospital Regensburg. Samples were plated on the corresponding selective media: Mueller Hinton for isolating Enterococcus and Mueller Hinton plates including 5% blood to isolate Streptococcus and Staphylococcus. One single colony of each bacterial strain was picked and cultivated overnight in 5 mL Lysogeny broth (LB) media at 37 °C and shaken at 220 rpm. Subsequently, 50 mL LB media was inoculated with 1 mL of the overnight culture and incubated for 4.5 h at 37 °C and 220 rpm. Then, each 1-L LB medium was inoculated with 7.5–10 mL bacterial suspension (depending on the growth rates of the bacterial strain) and incubated for 16 h at 37 °C and 150 rpm. Each strain was harvested during logarithmic growth phase by centrifugation at 4 °C for 10 min and 4000 rpm. The cell pellet was resuspended in 10 to 20 mL PBS, and cell lysates were produced by disruption in a French Press lysis at 1.5 k Bar and 3 °C yielding in 30 mL of the Streptococcus lysate at a concentration of 0.54 mg/mL, 30 mL of Enterococcus-lysate at a concentration of 0.62 mg/mL, and 30 mL of Staphylococcus lysate at a concentration of 1.42 mg/mL. After extraction, blood agar dishes were inoculated with aliquots of each lysate for 48 h. The absence of colony-forming units served to exclude the presence of remaining living bacteria. Total protein content of the lysates was determined in a Bradford Assay (BioRad Laboratories GmbH, Munich, Germany) and obtained lysates were aliquoted and stored at −20 °C.

### 4.4. Stimulation of UBMC with Gram-Positive Bacterial Lysates

Freshly isolated UBMC (2 × 10^5^) were resuspended in culture media (10% heat-inactivated human AB serum 1% ampicillin RPMI 1640 medium) and seeded in triplicate (90 µL per well) into polypropylene plastic 96-well plates (Nunc, Roskilde, Denmark). Noteworthy, the supplementation of culture media with the antibiotic ampicillin aimed to prevent the growth of potential residual live bacteria remaining after the French press procedure. Unless otherwise indicated, cells were stimulated with 0.157 prepared lysate in 100 µL final volume for 36 h at 37 °C in a humidified atmosphere containing 5% CO_2_. UBMC stimulated with PBS served as a negative control. Cell-free supernatants of stimulated cells were harvested by low-speed centrifugation (300× *g*, 10 min) and stored at −80 °C.

### 4.5. Determination of Cytokine and Chemokine Concentrations in UBMC Supernatants

The levels of cytokines and chemokines in the UBMC supernatants were determined applying the Luminex technology (MicroBIOMix GmbH, Regensburg, Germany). All the assays for a particular cytokine were run at the same time to limit intra-assay difference. First, the Human Cytokine 5-Plex panel (LHC6003, Invitrogen/ThermoFisher, Invitrogen Corporation, Carlsbad, CA, USA) and additional kits for detection of TGF-ß (LHG0121) and IL-5 (LHC0051) were employed to optimize the concentration of the lysate required. Subsequent Luminex assays were conducted using the following human singleplex beads kits (Invitrogen/ThermoFisher): IL-10 (LHC0101), IL-6 (LHC0061), IL-8 (LHC0081), IL-12 [p40/p70] (LHC0121), TNFα (LHC3011), IFNγ (LHC4031), MIP-1α(LHC1021) MIP-1β(LHC1051), and MCP-1 (LHC1011). Experiments were performed using the Human Extracellular Protein Buffer Reagent Kit (LHB0001, Invitrogen/ThermoFisher, Invitrogen Corporation, Carlsbad, CA, USA) according to the manufacturers’ protocol. The assays were performed in 96-well filter bottom plates. The beads were protected from light throughout the procedure. The lyophilized standard was reconstituted in 2 mL assay diluents and 1:3 serial dilutions were undertaken to generate a seven standard concentration set, while diluent alone served as a blank. The filtered plates were pre-washed with 200 µL of wash solution/well for 30 s. Wash solutions were aspirated using a vacuum manifold, and the bottom of the plate was blotted on paper towels to remove residual liquid. The concentrated bead mix was diluted 1:20 in wash solution. The bead solution was vortexed and sonicated immediately prior to adding 50 µL/well. The plate was washed twice with 200 µL wash solution/well as above. Incubation buffer (50 µL) and each standard (100 µL) were added in duplicate. Assay diluent (50 µL) was added to each well followed by the addition of the sample (50 µL). Given that in the optimization experiments (Figure 1) levels of IL-8, MCP-1, and MIP-1β exceeded the detection range of the Luminex assay in the concentration of 0.157 µg/mL of bacteria lysate, a 1:100 dilution of the supernatant was employed in the measure of these chemokines. The plate was covered and incubated for 2 h at RT on an orbital plate shaker (600 rpm). Afterwards, the liquid was removed using a vacuum manifold, and the plate was washed twice. A biotinylated detection antibody (100 µL) was added and plate was covered and incubated on the shaker for 1 h at RT. The plate was washed twice prior to the addition of Streptavidin-RPE (100 µL), then covered and incubated for 30 min at RT on the shaker. Finally, the plate was washed three times. The wash solution (110 µL) was added to each well, and the plate was covered, placed on the orbital shaker and incubated for 2–3 min at RT prior to analysis. Mean fluorescence intensity (MFI) was acquired using the Luminex xMAP 100 system (Luminex Corporation, Austin, Texas, USA) Software was set to acquire data using the 70 µL sample and to count 100 events per single bead set.

Cyto- and chemokine concentrations were calculated using a 4- or 5-parameter logistic fit curve generated from the 7 standards using the Liquichip Analyzer software (Liquichip™-Analyser-Software, Qiagen GmbH, Hilden, Germany) and expressed in pg/mL in accordance with international standards.

### 4.6. Statistical Analysis

Depending on the experimental design, statistical differences between measured values were analyzed either using nonparametric one-way ANOVA (Figure 1) or nonparametric Friedman test (Figure 2) for paired samples, followed by post hoc comparisons to detect differences between groups. *p* values ≤ 0.05 were considered statistically significant. Mann–Whitney U test was used to compare the levels of each cytokine for each bacteria lysate between UBMC cultures derived from female and male donors (Figure 3) and IL-8 levels in cord blood from preterm born neonates (Figure 4). Statistical tests were performed using SPSS 25 (for Windows) and GraphPad Prism 8.4.3.

## Figures and Tables

**Table 1:** Descriptive information about the UBMC donors.

Subject Number	Maternal Age (Years)	Parity	Gestational Age (Weeks + Days)	Maternal BMI Before Pregnancy	Neonatal Birth Weight (g)	Neonatalsex
1	31	6	40 + 3	25.9	3810	Male
2	29	2	41 + 0	24.1	3540	Female
3	33	1	39 + 4	31.9	4150	Female
4	29	2	40 + 6	30.8	3740	Male
5	27	1	40 + 1	22.1	3150	Female
6	34	1	40 + 5	35.0	3300	Male
7	25	2	38 + 5	32.4	3800	Male
8	27	1	39 + 4	22.5	3620	Male
9	30	2	37 + 1	21.5	2830	Female
10	27	1	37 + 0	22.9	2570	Female
**Median**	**29**	**1.5**	**39 + 3**	**25.0**	**3580**	
**Range**	**25–31**	**1–6**	**37–41**	**21.5–35.0**	**2570–4150**	

Abbreviations: BMI, body mass index.

## Data Availability

The data presented in this study are completely available in the present manuscript and supplementary figures.

## Supplementary Materials

The following are available online at https://www.mdpi.com/1422-0067/22/1/332/s1.
