# Supplementary material for: Perinatal Gram-Positive Bacteria Exposure Elicits Distinct Cytokine Responses In Vitro
Source: Int J Mol Sci. 2020 Dec 30;22(1):332. doi: 10.3390/ijms22010332 (PMC7795300; doi:10.3390/ijms22010332)
Supplement: Supplementary file 1 [file ijms-22-00332-s001.pdf]

Table S1. Storage time of umbilical cord blood from collection at delivery until isolation of UBMC.

Subject number	Time to umbilical cord blood processing (hours)
1	4
2	3
3	1
4	5
5	10
6	11
7	2
8	5
9	1
10	6
11	5
12	9
**Median**	**5.2**
**Range**	**1.0-11.0**

Table S2. Descriptive statistic information about the gestational age of non-infected (control) and infected preterm newborns. No significant differences were observed in the gestational age ($p > 0.05$, Mann Whitney U Test)

Preterm neonates		Non-infected	Infecfected
Number of samples		10	10
gestational age (days)	Median	242,5	222
	Minimum	198	170
	Maximum	258	254

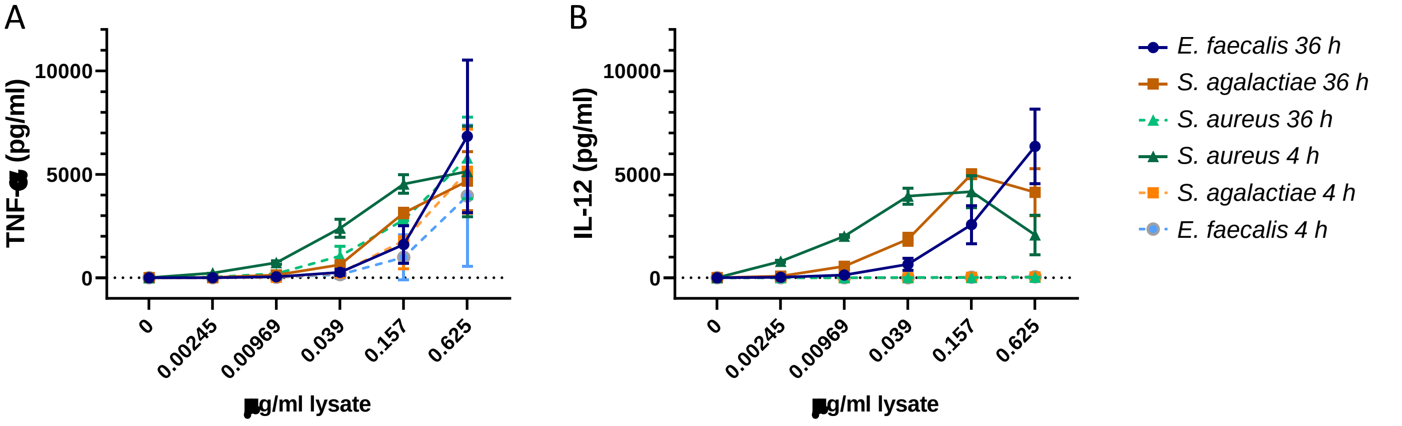

Figure S1. The culture of umbilical blood mononuclear cells with bacteria lysates for 36 h allows the detection of innate and adaptive cytokine responses. UBMC were co-cultured with media or with increasing concentrations of bacteria lysate (0.00245-0.625 µg/ml lysate) for 4 or 36 hours. Thereafter, the levels of the innate cytokines TNFα (A) and of the IL-12 (B) were determined using luminex technology. The effect of the time was compared in cultures stimulated with consecutive dilutions of the same bacteria. Although the levels of both cytokines were higher after 36 h than 4 h of culture, only in the case of IL-12 this difference reached statistical significance for all three bacteria investigated (Two-way ANOVA, factor: time, $p<0.05$).

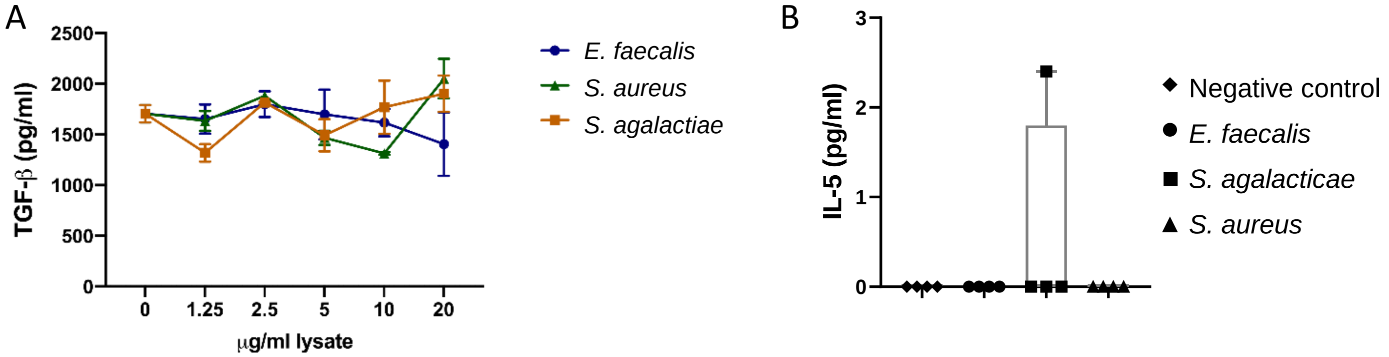

Figure S2. Co-culture of umbilical blood mononuclear cells and bacteria lysates did not significantly induced the secretion of TGFβ or IL-5. (A) No significant effect was observed for TGFβ after co-culture of UBMC with media or with increasing concentrations of bacteria extract (1.25-20 µg/ml lysate) for 36 hours (Two-way ANOVA, factor: bacteria lysate concentration, $p > 0.1$). (B) Similarly, incubation of UBMC with 0.157 µg/ml of the three bacteria lysate for 36 hours did not suffice to induce IL-5 secretion, that remained close to detection levels (One-way ANOVA, factor: bacteria lysate, $p > 0.1$).
